# Influence of the Mechanical Environment on the Regeneration of Osteochondral Defects

**DOI:** 10.3389/fbioe.2021.603408

**Published:** 2021-01-27

**Authors:** Sarah Davis, Marta Roldo, Gordon Blunn, Gianluca Tozzi, Tosca Roncada

**Affiliations:** ^1^School of Pharmacy and Biomedical Sciences, University of Portsmouth, Portsmouth, United Kingdom; ^2^Zeiss Global Centre, School of Mechanical and Design Engineering, University of Portsmouth, Portsmouth, United Kingdom

**Keywords:** osteochondral defects, tissue engineering, biomaterials, articular cartilage, mechanobiology, stem cells, mechanical testing

## Abstract

Articular cartilage is a highly specialised connective tissue of diarthrodial joints which provides a smooth, lubricated surface for joint articulation and plays a crucial role in the transmission of loads. *In vivo* cartilage is subjected to mechanical stimuli that are essential for cartilage development and the maintenance of a chondrocytic phenotype. Cartilage damage caused by traumatic injuries, ageing, or degradative diseases leads to impaired loading resistance and progressive degeneration of both the articular cartilage and the underlying subchondral bone. Since the tissue has limited self-repairing capacity due its avascular nature, restoration of its mechanical properties is still a major challenge. Tissue engineering techniques have the potential to heal osteochondral defects using a combination of stem cells, growth factors, and biomaterials that could produce a biomechanically functional tissue, representative of native hyaline cartilage. However, current clinical approaches fail to repair full-thickness defects that include the underlying subchondral bone. Moreover, when tested *in vivo*, current tissue-engineered grafts show limited capacity to regenerate the damaged tissue due to poor integration with host cartilage and the failure to retain structural integrity after insertion, resulting in reduced mechanical function. The aim of this review is to examine the optimal characteristics of osteochondral scaffolds. Additionally, an overview on the latest biomaterials potentially able to replicate the natural mechanical environment of articular cartilage and their role in maintaining mechanical cues to drive chondrogenesis will be detailed, as well as the overall mechanical performance of grafts engineered using different technologies.

## Osteochondral Defects

Osteochondral defects are areas of damage that involve both the articular cartilage and the underlying subchondral bone and can be caused by ageing, diseases (such as osteoarthritis and osteochondritis dissecans) or trauma. Osteoarthritis (OA) is a degenerative joint disease that affects over 250 million people worldwide (Hunter et al., 2014). Prevalence of the disease is increasing due to an ageing population and, in the US alone, 70 million people over the age of 65 are at risk of developing OA by the year 2030 (Bhatia et al., 2013). OA, originally thought to be a disease primarily affecting articular cartilage, is now considered to affect all tissues in the diarthrodial joint, including subchondral bone, ligaments, menisci, joint capsule, and synovial membrane (Torres et al., 2006; Hunter et al., 2009; Lo et al., 2009; Krasnokutsky et al., 2011). As well as changes to the chondrocytes and the cartilage matrix, osteoarthritis is characterised by structural changes such as joint space narrowing, osteophyte formation and subchondral sclerosis that cause pain and joint immobilisation. Subtle changes to subchondral bone can be observed early, though the precise chronology of how these changes affect the OA process remains to be uncovered and the role of the subchondral bone in initiating and advancing disease progression is now receiving greater attention (Li et al., 2013). The crosstalk between subchondral bone and articular cartilage is complex and can induce biomechanical and biochemical changes in the overlying cartilage (Hu et al., 2020). The more obvious effect of changes to subchondral bone can be seen in conditions such as osteonecrosis, osteosclerosis, and Osteochondritis Dissecans (OCD). In osteonecrosis, and osteosclerosis imbalances in the bone remodelling process causes changes in bone turnover, mineralization, and subchondral bone volume, reducing overall bone density. This alters the biomechanical environment of the osteochondral unit and causes strain changes in the overlying cartilage during loading that may lead to pathological changes. OCD is a pathologic condition that affects subchondral bone formation resulting in subchondral bone fragments that disrupt the overlying articular surface. The exact causes of OCD are still unknown, yet repetitive microtrauma, abnormal endochondral ossification, and genetic factors are thought to play a role in its development (Grimm et al., 2014). Primarily, repetitive overloading or trauma is thought to disrupt the blood supply resulting in osteonecrosis. This in turn, may induce microcracks in the subchondral bone plate and underlying bone, resulting in fragmentation of bone and cartilage, causing inflammation, and joint pain. Another example is where cartilage loss adjacent to subchondral bone marrow lesions is common and is probably associated with changes in the modulation of this crosstalk (Hunter et al., 2009).

Articular cartilage is a viscoelastic tissue that provides a smooth and lubricated surface for joint movement, which also plays a key role in the absorption and dissipation of loads to the underlying subchondral bone. Healthy articular cartilage is an avascular, a-neural and a-lymphatic tissue, composed mainly of a proteoglycan rich extracellular matrix (ECM), type II collagen and chondrocytes. Mechanical properties of articular cartilage largely depend on ECM composition and organisation, however, mechanical stimulation is essential for cartilage development as well as maintaining cartilage homeostasis (Sanchez-Adams et al., 2014; Prein et al., 2016). Nevertheless, it has been demonstrated that excessive loading, either as single acute event or repetitive stresses, induces the expression of degradative enzymes such as metallopeptidase with a thrombospondin type 1 motif 5 (ADAMTS5) and matrix metalloproteinase-13 (MMP13), affecting matrix composition and hence playing a pivotal role in pathogenesis (Nakagawa et al., 2012; Buckwalter et al., 2013; Houard et al., 2013; Chang et al., 2019). Both OA (particularly post-traumatic osteoarthritis: PTOA) and OCD are associated with high-impact sports and abnormal loading/ joint injury, and therefore tend to affect highly stressed joints such as the knee and elbow. Since mechanical loading plays such a vital role in the initiation and progression of osteochondral defects and associated conditions, a deeper understanding of cartilage-bone mechanics is essential for developing better diagnosis and treatment methods.

This review will focus on the biomaterials able to replicate the natural mechanical environment of articular cartilage and the effect of mechanical cues resulting from the use of these scaffolds in directing and enhancing chondrogenesis. Importantly, osteochondral implants must be able to withstand the mechanical environment in the joint, which is responsible for these mechanical cues and that they are tested during their initial development with this environment in mind.

## The Mechanical Environment of Natural Cartilage

**Figure 1:**
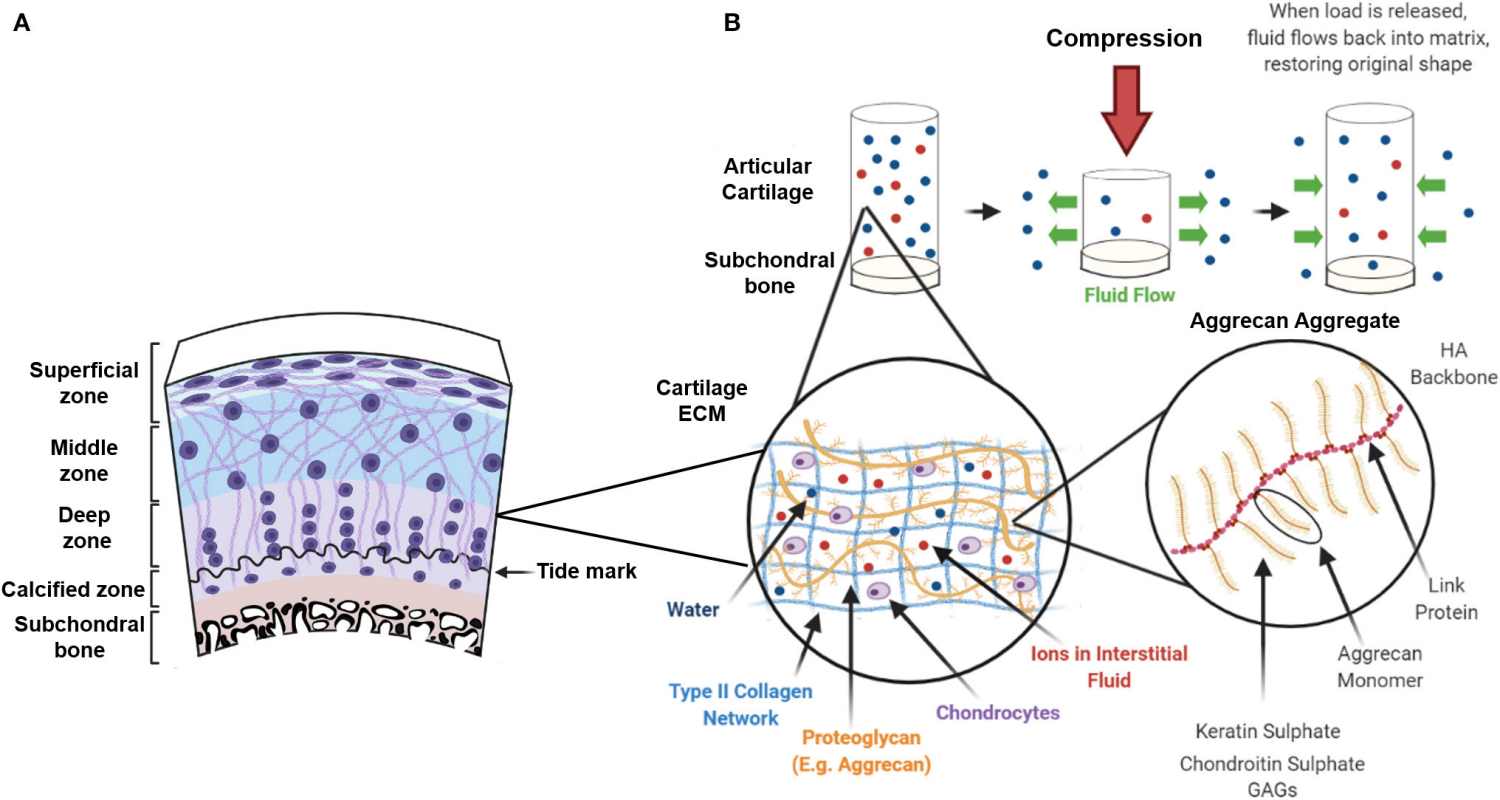
Zonal architecture of articular cartilage and viscoelastic behaviour following compression. **(A)** Articular cartilage can be subdivided into 4 distinct zones: The superficial zone, the middle zone, the deep zone and the zone of calcified cartilage. Collagen fibre orientation in the extracellular matrix (ECM) and distribution of chondrocytes varies within each zone. **(B)** The ECM consists of a liquid phase (the interstitial fluid) and a solid phase composed of a type II collagen network interwoven with proteoglycans (predominantly aggrecan). The negative charge on the keratin sulphate and chondroitin sulphate glycosaminoglycans (GAGs) attracts positive ions that creates an osmotic pressure and retains water in the collagen network. When a compressive load is applied fluid flows out of the ECM in a time-dependent manner and similarly, when the load is removed, fluid is drawn back into the matrix restoring its original shape.

Articular cartilage can be subdivided into four distinct zones: the superficial zone, the middle zone, the deep zone and the calcified zone (Figure 1A). Each zone exhibits a particular arrangement and organisation of chondrocytes and ECM proteins, mainly collagen type II (Col II) and proteoglycans, determining the tensile strength, flexibility and load-bearing ability of cartilage (Baumann et al., 2019). Since articular cartilage is a non-uniform structure, it presents challenges when trying to determine strain patterns and relative stiffness. This is due to variation in the orientation of collagen fibres, proteoglycan distribution, and molecular/ion content throughout the depth of native cartilage, which is a function of the anatomical location within the joint, and the type of loading applied.

The superficial zone represents the 10–20% of articular cartilage and contains flattened chondrocytes. In the superficial zone, thin collagen fibres (mainly collagen type II and IX) are tightly packed and aligned parallel to the articular surface to protect deeper layers from shear stress (Sophia Fox et al., 2009; Correa and Lietman, 2017). Moreover, the parallel arrangement of collagen provides tensile stiffness and strength providing the tissue with high mechanical stability. This thin layer acts as a barrier regulating not only the diffusion transport of nutrients and oxygen to the underlying cartilage structures but also the ingress and egress of large biomolecules (Leddy et al., 2008). Lubricin, which is responsible for reducing surface friction, is produced by chondrocytes only in this zone (Correa and Lietman, 2017). The middle zone represents 40–60% of the articular cartilage and is characterised by sparsely distributed rounded chondrocytes and a proteoglycan rich ECM (consisting mainly of aggrecan) (Fisher et al., 2019). Within the middle zone, collagen type II has thicker fibres and is obliquely distributed. The deep zone is characterised by the highest proteoglycan content and the lowest water concentration. Collagen fibres are thick and run perpendicular to the articular surface (Baumann et al., 2019). Chondrocytes are parallel to the collagen fibres and arranged in columns. Due to the high content of negatively charged proteoglycans, the deep zone is responsible for providing the greatest compressive resistance to articular cartilage (Gilbert and Blain, 2018). The deep zone is reported to have the highest stiffness, along with the superficial zone, corresponding to locations where the collagen fibre density is greatest, whereas the middle zone is characteristically softer (Schinagl et al., 1997; Bellucci and Seedhom, 2001). Articular cartilage stiffness has been reported to range from 0.1 to 6.2 MPa, with variabilities among studies that depend on sample type and testing setting (Boschetti et al., 2004; Robinson et al., 2016; Patel et al., 2019; Zheng et al., 2019; Guimarães et al., 2020). Within the middle and the deep zone, each chondrocyte is surrounded by a 2–4 μm thick collagen type VI rich pericellular matrix (PCM), which forms the chondron. The PCM seems to play a functional role in initiating signal transduction within the cartilage during load-bearing (Leddy et al., 2008). A study by McLeod et al. (2013) showed depth-dependent mechanical inhomogeneity of the elastic moduli of the ECM throughout the cartilage zones, yet zonal uniformity of the PCM elastic moduli in comparison. Cartilage stiffness has also shown to decrease with increasing severity of OA (Kleemann et al., 2005). The calcified zone is characterised by hypertrophic chondrocytes and has a high content of collagen type X (Col X). It anchors the collagen fibrils from the deep zone to the subchondral bone providing optimal integration and as it is infrequently penetrated by blood vessels it prevents vascularization of the articular cartilage. The zone of calcified cartilage also acts as a transitional zone and is important for reducing stress concentrations at the cartilage-bone interface (Boushell et al., 2017). The subchondral bone plate starts at the tidemark separating calcified and non-calcified cartilage. It is a supportive structure that consists of calcified cartilage and underlying subchondral bone that allows the build-up of hydrostatic pressure (Hwang et al., 2008). Damage to the integrity of the subchondral bone affects the generation of hydrostatic pressures and the repair of osteochondral defects often fails to recognise the importance of the subchondral bone plate. In its natural environment, cartilage is subject to a variety of different types of mechanical forces, including tension, compression, shear stress, and torsion. Physiological load on articular cartilage ranges from 5 to 8 MPa during walking and can reach up to 18 MPa when undergoing other activities such as rising from a chair (Clements et al., 2001). Due to the impermeable nature of the calcified cartilage and the low hydraulic permeability of the subchondral bone plate, the resistance to fluid flow within the cartilage results in the build-up of hydrostatic pressures (Hwang et al., 2008). Articular cartilage is resistant to these loads due, in part, to its viscoelastic behaviour resulting from the inter-relationship between the proteoglycan aggregates of the ECM (often referred to as the solid phase), and the interstitial fluid or liquid phase. The negatively charged carboxyl and sulphate groups of the proteoglycans attracts positive ions and creates an osmotic pressure, restrained by the tensile properties of the type II collagen network, which provides the cartilage with its compressive stiffness (Ateshian et al., 2004). When a constant force is applied, the interstitial fluid pressure increases, forcing fluid out of the porous ECM in a time-dependent manner, creating frictional drag until equilibrium is reached. This frictional drag is inversely proportional to its permeability (Mak, 1986) and gives the cartilage its viscoelastic creep and stress-relaxation characteristics during compression (Mow et al., 1980; Halonen et al., 2014). When strain is kept constant, stress on the tissue increases until it reaches a peak which, due to redistribution of fluid within the cartilage, relaxes over time until equilibrium is reached. Similarly, when the load is removed, fluid flows back into the matrix allowing the cartilage to return to its original state, hence giving the tissue its mechanical properties and ability to withstand compressive loads (Figure 1B). Structural and biochemical variations relating to degenerative changes following injury or pathological conditions such as OA, alters the fluid flow dynamics throughout the tissue and can further affect load-bearing and compressive capability.

Cartilage was originally described as a biphasic material by Mow et al. (1980), composed of the liquid and solid phases as previously described. However, the model was adapted into a triphasic material by Lai et al. (1991) to include the mechano-electrochemical behaviour of monovalent ions and later the model accounted for the polyvalent ions in the interstitial fluid as forces acting as part of a separate liquid or ion phase (Gu et al., 1998). Although the triphasic model is a more recent theory that encompasses a structurally more accurate description of the composition of articular cartilage, the biphasic model highlights the importance of osmotic and hydrostatic pressure within the cartilage and how the tissue resists both compressive and tensile forces (Ateshian et al., 2004). It should be noted that any successful osteochondral implant has to accommodate these forces.

### The Effect of Physiological Loading, Overuse, and Disuse on Articular Cartilage

The high and complex range of physiological loads applied to cartilage are critical for maintaining healthy joint function. Mechanical loading, in the form of moderate exercise, is one of the most important factors for maintaining a homeostatic environment and balancing the anabolic and catabolic response of chondrocytes for ECM synthesis and degradation. Numerous studies have shown reduction in pro-inflammatory cytokines (IL-1B, IL-6 TNF-α), inflammatory mediators (COX-2, PGE_2_ and NO) (Chowdhury et al., 2001; Fu et al., 2019) and reduction in matrix-degrading enzymes (MMPs and ADAMTSs) in response to dynamic compression (Sun et al., 2012). *In vitro* studies also confirm anti-inflammatory effects of loading, with an increase in both gene expression, synthesis of type II collagen, aggrecan production (Buschmann et al., 1999; Waldman et al., 2006; Iseki et al., 2019) and stimulation of chondrocyte growth, differentiation, and proliferation. It is also important to note that chondrocytes from different regions of cartilage constitutively express mRNA for cartilage structural proteins in different baseline levels and respond differently to mechanical loading, suggesting that isolating chondrocytes from a non-load-bearing area might significantly affect the quality of the synthesised ECM (Bevill et al., 2009; Briant et al., 2015).

**Figure 2:**
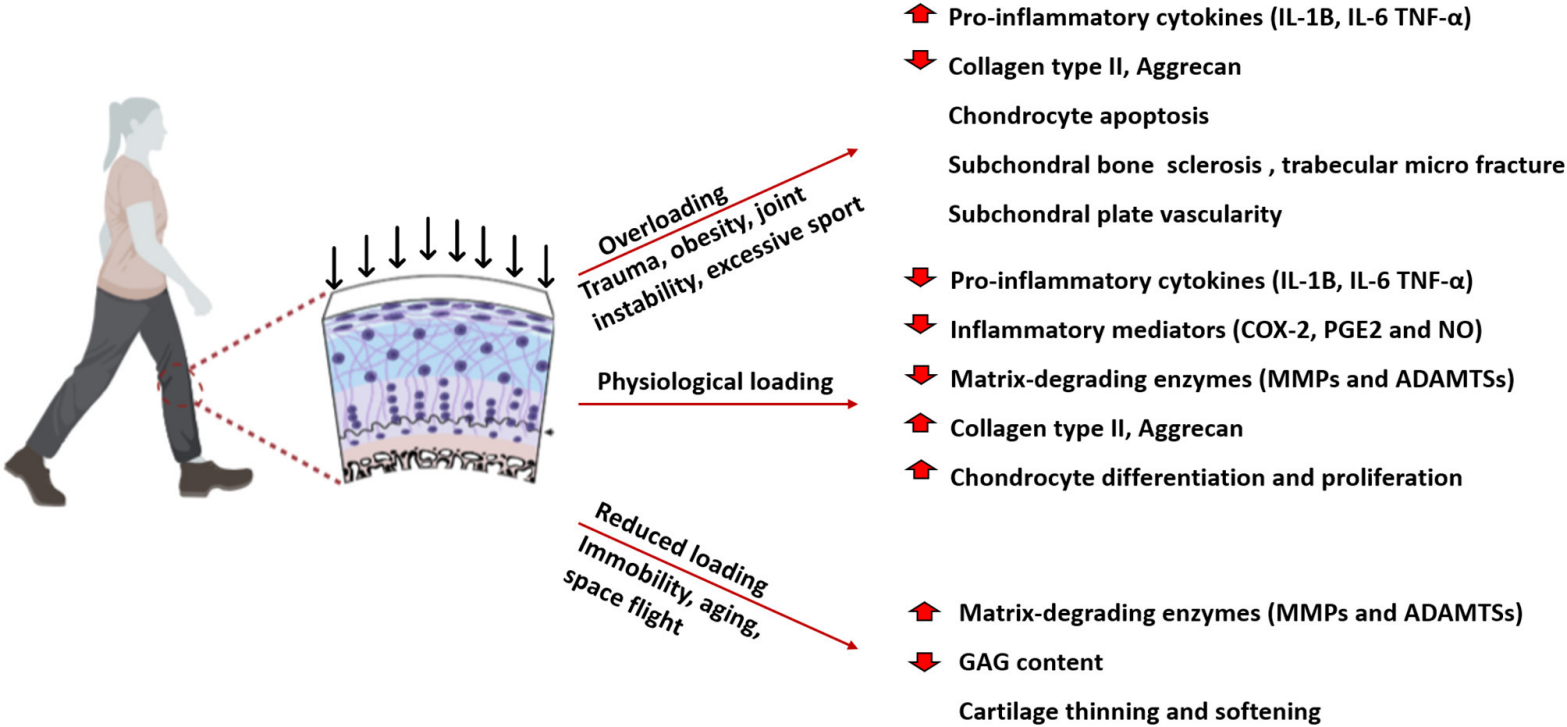
Schematic representation of the effect of physiological, overloading, and reduced loading on articular cartilage. Physiological loading is essential for maintaining cartilage homeostasis regulating ECM synthesis and chondrocytes proliferation. Overloading, caused by trauma, obesity and joint instability, reduces collagen, and aggrecan content inducing chondrocytes apoptosis. Reduced loading increase matrix degradation leading to cartilage thinning and softening.

Although there is a genetic predisposition to the development of OA, loading plays a contributory role. Physiological loading is important for maintaining joint homeostasis (Figure 2), whilst abnormal loading caused by obesity, immobilisation, joint instability, overuse, or trauma can cause cartilage degradation and are the main risk factors linked to the development of OA (Arden and Nevitt, 2006). Overloading of the joint, either as a single impact load or cyclic loading causes increased catabolism, chondrocyte necrosis and apoptosis and damage to the collagen network in a dose-dependent manner (Chen et al., 2001; Clements et al., 2004; Hosseini et al., 2014). Most studies report a critical threshold with chondrocyte apoptosis, GAG loss and increased production of inflammatory cytokines above this threshold load (Clements et al., 2001; D'Lima et al., 2001). Kerin et al. (1998) indicated that loads above 10 MPa can result in apoptosis. In comparison, using bovine explants, Loening et al. (2000) showed that chondrocyte apoptosis can occur at 4.5 MPa as an earlier response to injury which is later followed by degradation of the collagen network at 7–12 MPa (Loening et al., 2000). On the other hand reduced mobility, which is associated with low loading conditions results in upregulation of MMPS, softening and a reduction in proteoglycan content and cartilage thinning (Jurvelin et al., 1986; Vanwanseele et al., 2002; Leong et al., 2011). Impaired joint loading significantly affects articular cartilage ECM composition and as consequence cartilage becomes thinner with reduced ability to absorb loads and shocks resulting in excessive load transmission to the underlying subchondral bone. Abnormal mechanical load can induce bone marrow oedema and subchondral sclerosis (Beckwée et al., 2015; Eriksen, 2015; Donell, 2019).

Articular cartilage is not only sensitive to the type of force applied and the magnitude of load but also to the duration, direction, and frequency of loading (Komeili et al., 2019). Párraga Quiroga et al. (2017) showed that higher strain rates cause more damage to the collagen network, while lower strain rates cause more damage to the non-fibrillar matrix components and that overall cartilage damage is both load and rate dependent. A study by Sadeghi et al. (2015) showed increases in crack length and surface damage with increasing loading frequency above a normal level of 1 Hz. There may also be variation in the material properties of the articular cartilage, in that weight-bearing areas may be more functionally prepared for loading compared to non-weight-bearing areas, and that non-weight-bearing areas may be more susceptible to damage and fibrillation when subject to the same tribological stresses (Moore and Burris, 2015). These factors highlight variability and therefore difficulty for a standard osteochondral graft material to be able to replicate native cartilage in different regions and locations within the same joint, let alone variability between different joints and that under different loading conditions.

### Variations in Strain and Stiffness

In native osteochondral tissue under normal loading conditions, cartilage can experience strains of 2–9% and can reach up to 20–30% during vigorous activity, whereas the underlying bone experiences strains of <1% (Sanchez-Adams et al., 2014; Steinmetz et al., 2015). In addition, calcified cartilage is ~100 times stiffer than hyaline cartilage and 10 times less stiff than underlying subchondral bone and this transitional zone plays a crucial role in the transmission of loads between these regions (Mente and Lewis, 1994; Madi et al., 2020).

Strain distribution patterns vary depending on the type of loading, with more uniform strains during dynamic loading than static loading conditions and tension-compression non-linearity also causes variations in tensile stiffness (Huang et al., 2005; Komeili et al., 2019). Since cartilage is anisotropic material, the tensile moduli varies depending on the direction of testing and shows increased stiffness parallel to the local split-line patterns which also varies throughout the cartilage depth (Kempson et al., 1973). Variations have also been reported between anatomical locations within the joint, and differences in tensile modulus have been observed between high and low weight-bearing regions. For example, Wong and Sah (2010) showed regional variations in tibial and femoral cartilage, with more axial strain present in tibial cartilage during joint articulation. Several studies have also reported that in tibial and femoral knee condyle, higher strains are present on the medial side compared to the lateral compartment which provides an explanation for differences in mechanical stiffness and is related to contact biomechanics at these sites (Liu et al., 2010; Cotofana et al., 2011; Coleman et al., 2013; Halonen et al., 2014).

Asymmetric strain patterns of natural cartilage create numerous challenges for tissue engineers when analysing strain distribution throughout the cartilage and for replicating this mechanical environment *in vitro* in order to enhance the maturation of the tissue engineered construct. However, mechanical stimulation has successfully proven to enhance the properties of tissue engineered osteochondral grafts which will be discussed in more detail in this review. It is important to note that *in vivo* the complex interplay of other supportive tissues such as the menisci, tendons and ligaments (Halonen et al., 2014) which may also be compromised by trauma and OA.

## Current Clinical Therapies

Due to its avascular nature, the lack of abundant nutrients and low cell density, cartilage has limited regenerative capacity (Lo Monaco et al., 2018; Medvedeva et al., 2018). The treatment modality for repairing osteochondral injuries is dependent on the depth and area of the defect. Several clinical treatments are available to treat osteochondral defects such as microfracture (marrow stimulation), and the use of osteochondral autografts and allografts (Nukavarapu and Dorcemus, 2013; Freitag et al., 2017; Mathis et al., 2018). Microfracture is a surgical technique used to treat chondral defects, it involves perforating the subchondral bone with tiny holes allowing bone marrow mesenchymal stem cells and biomolecules to infiltrate the defect (Erggelet and Vavken, 2016). However, this often promotes the formation of mechanically inferior fibrocartilage with little evidence of type II collagen deposition (Redondo et al., 2018). For the treatment of larger osteochondral defects, where subchondral bone damage is seen tissue grafts of both cartilage and bone may be used. Osteochondral autograft transfer and mosaicplasty, have been used to treat full-thickness defects up to 4 cm^2^. During this procedure, chondral defects are replaced with plugs of the patient's own healthy articular cartilage and bone that are harvested from non-weight-bearing areas and transferred to pre-drilled holes at the defect site (Rowland et al., 2019). The outcome depends on age, sex and size of the lesion. In the case of large lesions, up to 8–9 cm^2^, multiple plugs can be used but with a risk of significant donor site morbidity (Richter et al., 2016; Kato et al., 2018). Unlike autograft, allografts use full-thickness cartilage that can be harvested from locations that correlate with the defects to be filled allowing more precise matching of the size and contour of the articular surface (Assenmacher et al., 2016; Haber et al., 2019). Even though allografts can be performed as a single staged procedure and have shown good survival rate in short to medium term (5–10 years), long term follow-up has shown considerable reoperation (30.2%) and high failure rates (18.2%) over time (Familiari et al., 2018). Moreover, allografts are limited by the lack of tissue supply, low cell viability due to graft storage and possible immunorejection (Yang et al., 2017; Mathis et al., 2018).

Among the current clinical therapies, multi-layered cell-free scaffolds have been considered and are currently under pre-clinical and clinical evaluation. TruFit CB (Smith and Nephew) is a synthetic plug designed to be used with microfracture in order to improve the mechanical stability of the defect. Initial studies showed positive results with the regeneration of cartilage in a goat model, however, clinical studies revealed that 70% of patients required reoperation and the plug failed specifically in restoring the subchondral bone (Williams and Gamradt, 2008; Joshi et al., 2012). The bone-layer of TruFit CB is made of PGA and calcium phosphate, two materials that degrade quickly post-implantation and mechanical failure has resulted in the plug being withdrawn from the market (Fraser et al., 2016; Tseng et al., 2020). D'Ambrosi et al. (2019), investigated the clinical and radiological efficacy of MaioRegen, a try-layered collagen-based scaffold, in restoring osteochondral knee defects. Despite the promising satisfactory and reliable results at mid-term follow-up, this systematic review revealed that, in terms of clinical improvement at follow-up, MaioRegen is not superior to conservative treatment or other cartilage techniques. Therefore, there is still an unmet need for an optimal biomaterial system that favours simultaneous bone and cartilage regeneration. Although current clinical approaches can reduce pain and improve the quality of a patient's life, none of them has routinely achieved complete healing of the osteochondral lesion. Non-biological man-made materials can be used to partially replace the joint (e.g., unicompartmental knee replacement) or when the whole joint is severely affected it is likely that a total joint replacement (TJR) will be required as an end-stage intervention. In the elderly TJR is a successful end stage treatment for OA however, younger patients have a significantly higher risk of undergoing revision due to implant limited lifespan (25 years), periprosthetic joint infection or aseptic mechanical failure (Meehan et al., 2014; Stambough et al., 2014; Bayliss et al., 2017; Evans et al., 2019). To overcome these limitations, in the last two decades, research has focused on tissue engineering (TE) as a possible solution for osteochondral regeneration and repair of cartilage.

## Tissue Engineering Approaches

The most common tissue engineering approach involves the use of a biocompatible scaffold, cells (e.g., stem cells) and/or a combination of bioactive molecules such as growth factors and cytokines. Autologous chondrocyte implantation (ACI) is a procedure for the regeneration of cartilage introduced by Brittberg et al. (1994), where autologous chondrocytes are isolated from a non-load-bearing site of the cartilage, expanded *in vitro* for 4–6 weeks and subsequently injected under a periosteal flap that is sutured onto the cartilage positioned over the defect (Könst et al., 2012). Although this technique has been used for two decades with successful surgical outcomes, the main issue is that two operations are required, one to obtain the cells, using arthroscopy and the other usually an open procedure to implant the cells (Minas et al., 2014; Mistry et al., 2017; Zikria et al., 2019). Matrix-induced autologous chondrocytes implantation (MACI) was originally developed to improve the biological performance of autologous chondrocytes cells and simplify surgical procedures (Andriolo et al., 2020). As with ACI, chondrocytes are isolated from a non-load-bearing area and cultured *in vitro*, however, this approach aims to deliver autologous chondrocytes in a biopolymer membrane. MACI® is also the name of a commercially available membrane of porcine collagen type I/III (Genzyme, United States). Several types of membranes and scaffolds have been developed for MACI procedures such as Novocart®3D (TETEC Tissue Engineering Technologies AG, Germany) a collagen-chondroitin-sulphate based membrane, CaRes®–Cartilage Regeneration System (Arthro-Kinetics, Germany) a collagen type I matrix and Cartipatch® (Tissue Bank of France, France) a monolayer agarose-alginate hydrogel (Vilela et al., 2018). However, MACI failed to prevent fibrocartilaginous healing and the integration of the scaffold into host hyaline cartilage is still unsatisfactory due to the intrinsic features of fully differentiated chondrocytes with their poor capability of tissue remodelling. Moreover, MACI still requires a two-step surgery, cartilage biopsy and cell cultivation, thus increasing the total cost (Behrens et al., 2006; Zikria et al., 2019). To further improve ACI outcomes and obtain a more reliable tissue repair, third generation of ACI has been developed, in which autologous chondrocytes are cultured in 3D to form spherical aggregates with a self-synthesised extracellular matrix. Spheroids of human autologous matrix-associated chondrocytes (Spherox) is an advanced tissue medicinal product with European Medicines Agency (EMA) market approval for the treatment of osteochondral defects up to 10 cm^2^ (Niemeyer et al., 2020). However, due to differences in cartilage phenotype isolating chondrocytes from a non-load-bearing area might significantly affect the quality of the synthesised ECM (Bevill et al., 2009; Briant et al., 2015).

So far there appears to be little difference in outcomes of these cell therapies and tissue engineering approaches when compared with osteochondral autograft transfer system, mosaicplasty or microfracture surgery. Further, when harvested *in vitro*, chondrocytes undergo dedifferentiation exhibiting a flattened, fibroblast-like morphology. In these conditions they produce a higher amount of collagen type I and collagen type X inducing the formation of fibrocartilage. An advantage of growing spheroids of chondrocytes isolated from biopsies is that the cartilage phenotype is better maintained than when cells are grown on flat tissue culture plastic. However, all of these approaches fail to fully repair the lesion in severe osteochondral defects, where both subchondral bone and articular cartilage are damaged (Davies and Kuiper, 2019). A significant proportion of research is focusing on the use of stem cells for cartilage repair since a large number of cells can be obtained from different sources such as bone marrow, peripheral blood, adipose tissue, dental pulp, placenta, and the umbilical cord (Tozzi et al., 2016). However, selectively promoting stem cell differentiation into appropriate cell lineages *in situ* is still challenging. An expanding field of research has demonstrated that mechanical cues from the environment could drive tissue formation and maturation, suggesting that combining scaffolds with mechanical properties that can drive stem cell differentiation could provide a solution for osteochondral defects where both bone and cartilage formation is required.

## Osteochondral Grafts Materials That Can Be Used to Replicate the Natural Mechanical Environment

The development of an osteochondral implant that replicates the structure of articular cartilage and subchondral bone remains challenging for tissue engineers. Material selection plays a pivotal role in the development of osteochondral grafts as it potentially contributes to the mechanical properties of the scaffold (Jahr, 2017; Bonani et al., 2018).

### Natural Materials

Natural materials such as collagen, chitosan, hyaluronic acid, silk, and alginate have been extensively used in TE for their biocompatibility, degradability and bioactivity (Jeuken et al., 2016; Li et al., 2018b). Natural materials are often used in the form of hydrogels with a highly hydrated viscoelastic matrix, tunable swelling behaviour and mechanical properties depending on the type and degree of crosslinking (Catoira et al., 2019; Mantha et al., 2019). Moreover, natural materials provide multiple binding sites for cell-ECM interaction. Multiple scaffolds for osteochondral TE in the clinical market are mainly composed of collagen type I (NOVOCART^®^3D, MACI^®^, CaReS^®^, NeoCart^®^, Maioregen^®^) (Kon et al., 2009; Crawford et al., 2012; Petri et al., 2013; Saris et al., 2014; Zak et al., 2014). Collagen can be extracted from various tissues and sources, for example, studies have reported that purified collagen can be isolated from vertebrate (generally rat, bovine, porcine and sheep) skin, tendon, cartilage and bone as well as from marine invertebrates (jellyfish, sponges, octopus, squid, cuttlefish, starfish) (Barzideh et al., 2014; Langasco et al., 2017). Even though collagen type I does not represent the main component of articular cartilage, several studies have demonstrated its pro-chondrogenic effects (Calabrese et al., 2017a,b; Xia et al., 2018). Preference of type I collagen in TE is largely attributed to its availability, its general biocompatibility and safety approvals granted by various agencies; however, high production costs and poor mechanical properties of pure collagen scaffolds are still major limitations (Table 1) (Dong and Lv, 2016; Ghodbane and Dunn, 2016). In comparison, Gelatin is derived by thermal denaturation of collagen and can be manufactured at a much lower cost and in larger quantities (Grover et al., 2012). Gelatin shows low antigenicity, it possesses integrin-binding sites, and it is completely resorbable *in vivo*. However, at body temperature gelatin hydrogels are not stable, limiting their possible use as a biomaterial. Van Den Bulcke et al. (2000) first described gelatin methacrylate (GelMA), a chemically modified form of gelatin that can be stabilised through photo-crosslinking allowing the formation of a hydrogel that is stable at body temperature. The modulus of GelMA-based biomaterial can be controlled by varying the degree of substitution and macromer concentration (Sadeghi et al., 2017), for example, Gan et al. (2019) has modified GelMA hydrogels by intercalating oligomers of dopamine methacrylate obtaining flexible hydrogels with compressive modulus of 2.5 MPa and shape-recovery ability. GelMA has also been used in combination with hyaluronic acid (HA), which forms the backbone of aggrecan and therefore plays a critical role in maintaining the viscoelastic and mechanical properties of cartilage (Hemmati-Sadeghi et al., 2018). HA acts also as a biochemical cue enhancing chondrogenic differentiation of MSCs, promoting chondrocyte proliferation and preventing chondrocyte de-differentiation by activating CD 44 (Chen et al., 2018; Li et al., 2018a; Yamagata et al., 2018). The use of HA in TE affects matrix deposition by cells, thus enhancing the dynamic and equilibrium moduli during *in vitro* culture (Levett et al., 2014). Recently silk fibroin (SF) has also been investigated in the context of osteochondral TE due to its biocompatibility, low immunogenicity, slow degradation rate, and remarkable mechanical properties (Qi et al., 2017). Silk has a high tensile strength (around 300–740 MPa) and depending on the source and production method, it is possible to obtain elastic moduli ranging from 1 MPa to 17 GPa, making it a favourable biomaterial not only for cartilage repair but also for subchondral bone (Koh et al., 2015; Peng et al., 2019). Li J. J. et al. (2015), developed a bi-layered scaffolds for osteochondral regeneration using silk fibroin for the cartilage layer and a silk-coated strontium-hardystonite-gahnite ceramic scaffold for the bone layer. The silk layer exhibited highly elastic behaviour showing 91% strain at failure, indicating that the silk scaffold could stretch to approximately twice its original length before breakage, which is desirable for the cartilage phase. When tested under compression the biphasic scaffold approximated the biomechanical behaviour of osteochondral tissue, as it could maintain structural integrity under large compressive stresses while retaining the ability for shape recovery when hydrated, in addition the stiff bone phase could withstand large compressive stresses with minimal deformation.

**Table 1:** Summary of advantages, disadvantages, and mechanical properties of naturally-derived materials.

**Natural materials**	**Advantages**	**Disadvantages**	**Mechanical properties**	**References**
Collagen type I	Low immunogenicity Degraded *in vivo* by MMPs	High production cost Low mechanical properties	Permeability 0.044–0.072 mm4/Ns Compressive modulus 3.5–3.7 kPa	Ghodbane and Dunn, 2016
Gelatin	Manufactured at a lower cost and in large quantities Low antigenicity Resorbable	Not stable at body temperature	Compressive modulus 0.75–6 kPa	Chen S. et al., 2016
GelMA	Stabilised form of gelatin Photocrosslinkable Varying the degree of substitution is possible to vary mechanical properties	UV crosslinking may have a negative effects on encapsulated cells	Compressive modulus 2–30 kPa	Sadeghi et al., 2017 Klotz et al., 2016
Hyaluronic acid (HA)	Enache MSCs chondrogenic differentiation Maintaining viscoelastic and mechanical properties in native cartilage Can be physically and chemically modified	Rapid degradation and poor mechanical properties	Elastic modulus of modified HA 1–70 kPa	Chen C. H. et al., 2016; Li et al., 2018a; Yamagata et al., 2018 Lee et al., 2018 Trombino et al., 2019
Silk fibroin	Biocompatibility Low immunogenicity Slow degradation rate Remarkable mechanical properties	Brittleness and swelling behaviour limits its applications in tissue engineering	High tensile strength 300–700 MPa and elastic modulus ranging from 1MPa to 17GPa	Koh et al., 2015; Chen et al., 2018; Peng et al., 2019
Alginate	Biodegradable Biocompatible Re-differentiate chondrocytes after monolayer culture Support chondrogenic phenotype Tunable mechanical properties	Lack of adhesion ligands	Elastic modulus 0.15-0.55MPa	Kaklamani et al., 2014
Chitosan	Biocompatibility Biodegradability Antibacterial properties	Display poor mechanical properties	0.13–0.199 MPa	Thomas et al., 2017

Among natural polysaccharides, both alginate and chitosan have potential for cartilage repair (Xu et al., 2008; Yao et al., 2016; Ewa-Choy et al., 2017; Henrionnet et al., 2017; Merlin Rajesh Lal et al., 2017; Ruvinov et al., 2018; Huang et al., 2019). Alginate is a biodegradable and biocompatible material, derived from seaweed that is composed of α-*D*-mannuronic acid and β-l-glucuronic acid. Studies have shown that it can support chondrogenic phenotype promoting a rounded morphology of isolated chondrocytes and the synthesis of type II collagen and proteoglycans (Homicz et al., 2003; Caron et al., 2012; Angelozzi et al., 2017; Aurich et al., 2018). Chondrogenic differentiation of stem cells isolated from bone marrow, adipose tissues, Wharton's Jelly, and dental pulp has been promoted by growing cells within alginate gels (Huang et al., 2015; Reppel et al., 2015; Ewa-Choy et al., 2017; Mata et al., 2017; Baba et al., 2018). Although much lower than the compressive modulus of native cartilage the mechanical properties of alginate scaffolds can be modified to give values of 0.15–0.55 MPa using divalent ions (Mg^2+^, Ca^2+^, and Sr^2+^) (Kaklamani et al., 2014). However, the main limitation of alginate-based materials is the lack of adhesion ligands that are essential for cell-attachment and to overcome this, bioactive components such as collagen may be incorporated (Bian et al., 2011; Lee and Mooney, 2012; Ganesh et al., 2013).

Another natural polymer employed is chitosan, derived from partial deacetylation of chitin, used in TE for its biocompatibility, *in vivo* degradation and antibacterial properties (Cheung et al., 2015; Varun et al., 2017; Huang et al., 2019). Chitosan hydrogels have been shown to support the proliferation of chondrocytes and MSCs *in vitro* and to improve the deposition of cartilaginous ECM both *in vitro* and *in vivo* (Griffon et al., 2006; Elder et al., 2013; Faikrua et al., 2013; Sheehy et al., 2015; Huang et al., 2019; Scalzone et al., 2019). However, since chitosan display poor mechanical properties, crosslinking or combination with other materials is required to optimise the elastic modulus for osteochondral TE (Muzzarelli et al., 2015; De Mori et al., 2019; Kusmono and Abdurrahim, 2019; Scalzone et al., 2019). Thomas et al. (2017) tuned the stiffness of chitosan-hydrogels by blending increasing concentrations of hyaluronic acid dialdehyde and the degree of crosslinking to obtain hydrogels with a Young's modulus of 0.13 MPa and 0.199 MPa. However, a reinforced chitosan-based scaffold failed to regenerate bone and cartilage *in vivo* suggesting that the crosslinking treatment may have affected its overall degradation (Roffi et al., 2019). Therefore, a careful balance between the mechanical properties and degradation rate should be considered when designing osteochondral scaffolds using this material.

### Synthetic Materials

Synthetic materials are attractive substitutes for load-bearing tissues, since the mechanical properties can be tailored by altering the molecular weight and/or via the use of different processing methods (Grigore, 2017). Synthetic polymers, including poly(ethylene glycol) (PEG), polylactide (PLA) and its derivatives poly(L-lactide) (PLLA) and poly(lactic-co-glycolic acid) (PLGA), polyglycolic acid (PGA), poly(ε-caprolactone) (PCL) and poly(vinyl alcohol) (PVA), are used to form hydrogels, porous scaffolds and nanofibrous scaffolds (Sánchez-Téllez et al., 2017; Yang et al., 2017; Castilho et al., 2018; Dai et al., 2018; Kudva et al., 2018; Critchley et al., 2020). The main disadvantage of these materials is the lack of specific binding motifs for cell attachment, but this can be improved through functionalization or by combining with more bioactive materials. PEG hydrogels have been used in TE due to their high solubility in water, hydrophilicity, biocompatibility, inertness, and non-immunogenicity (Table 2). They have also shown to maintain cell viability and promote chondrogenic ECM synthesis (Bryant and Anseth, 2002). By varying the molecular weight and the concentration of PEG precursors, Nguyen et al. (2012) obtained hydrogels with equilibrium modulus (0.01–2.46 MPa), hydraulic permeability [ranging from 10^−13^ to 10^−16^ (m^2^/Pa s)] and tensile modulus (0.02–3.5 MPa) similar to articular cartilage. Steinmetz et al. (2015) also developed a multi-layer PEG hydrogel resembling the zonal organisation of the osteochondral tissue. Although the compressive modulus did not match that of the native cartilage and bone when subject to mechanical loading, the strain distribution pattern was similar to osteochondral tissue with higher strain in the cartilage-like layer. When 7.5% apparent strains were applied to the hydrogel the local strains in the cartilage-like layer and in the bone-like layer were 15 and 2% respectively.

**Table 2:** Summary of advantages, disadvantages, and mechanical properties of synthetic materials.

**Synthetic materials**	**Advantages**	**Disadvantages**	**Mechanical properties**	**References**
PEG	High solubility in water Hydrophilicity Biocompatibility Inertness Non-immunogenicity	Lack of specific binding motifs for cell attachment	Equilibrium modulus 0.01–2.46 MPa Hydraulic permeability 10–13–10–16 m2/Pa Tensile modulus 0.02–3.5 MPa	Nguyen et al., 2012 Zhu, 2010
PGA	Biocompatible Bioresorbable	Loses its mechanical integrity between 2–4 weeks *in vivo*	Tensile modulus 7 GPa	Woodard and Grunlan, 2018; Gorth and Webster, 2011
PLA	PLA is more hydrophobic compared to PGA, leading to a slower hydrolysis rate.	Lack of specific binding motifs for cell attachment	Tensile modulus 3 GPa Tensile strength 50–70 MPa	Narayanan et al., 2016 Samavedi et al., 2013
PLGA	Modulation of Young's modulus and degradation rate, Sustained mechanical integrity after implantation	Lack of specific binding motifs for cell attachment	Compression storage modulus 3.2–4.6 MPa	Baker et al., 2009 Gentile et al., 2014
PVA	Biodegradable Biocompatible Adjustable mechanical properties	Lack of specific binding motifs for cell attachment	Tensile strength 1–17 MPa Elastic modulus 0.0012–0.85 MPa Low friction coefficients (μ) 0.02–0.05	Lin et al., 2017 Teixeira et al., 2019 Sánchez-Téllez et al., 2017
PCL	Adjustable mechanical strength Possibility to produce hydrogel, porous scaffold, electrospun nanofibers	Lack of specific binding motifs for cell attachment	Compressive modulus 6.63–56.46 MPa Tensile Modulus 6.03–46.04 MPa	Olubamiji et al., 2016

PGA exerts high tensile modulus (7 GPa) but due to its relatively hydrophilic nature and instability in aqueous solution loses its mechanical integrity between two and four weeks *in vivo* (Gunatillake and Adhikari, 2003; Gorth and Webster, 2011; Woodard and Grunlan, 2018). PLA exists in several isoforms and the presence of one extra methyl group makes it more hydrophobic compared to PGA, leading to a slower hydrolysis rate. PLA possesses a high tensile modulus (3 GPa) and strength (50–70 MPa) (Gorth and Webster, 2011; Samavedi et al., 2013). PLGA can be synthesised using a different ratio of PGA and PLA that allows modulation of both Young's modulus and the degradation rate which can be, from a few weeks up to months, resulting in sustained mechanical integrity after implantation (Félix Lanao et al., 2013; Samavedi et al., 2013; Gentile et al., 2014). PVA is a biodegradable and biocompatible polymer, from which hydrogels can be prepared at different polymer concentrations to obtain tensile strengths in the cartilage range of 1–17 MPa as well as an elastic modulus up to 0.85 MPa (Karimi and Navidbakhsh, 2014; Lin et al., 2017; Teixeira et al., 2019). PVA hydrogels exhibit limited swelling when tested at osmotic pressures similar to that of articular cartilage, which is desirable for soft tissue engineering to preserve the initial size and shape and to prevent interfacial debonding (Holloway et al., 2011; Oliveira et al., 2019). A non-biodegradable PVA based hydrogel (Cartiva^®^) exerts biphasic behaviour similar to normal articular cartilage under compression and it is currently under clinical trial for first metatarsophalangeal joint hemiarthroplasty (Brandao et al., 2020).

PCL is an FDA approved biodegradable aliphatic linear polyester and it is one of the most investigated polymers for tissue engineering applications due to its adjustable mechanical strength. PCL can be used to produce porous scaffolds as well as electrospun nanofibers (Zhu et al., 2014; Panadero et al., 2016). Visser et al. (2015) incorporated PCL microfibers into GelMA obtaining reinforced hydrogels with mechanical properties similar to articular cartilage. Castilho et al. (2019) also used PCL to successfully develop a bi-layered construct that mimics the zonal structure as well as the functional properties of native cartilage. This construct incorporated a thin superficial tangential layer, mimicking the collagen organisation in the superficial layer of the cartilage, that improved the load-bearing properties of the micro-fibre reinforced hydrogel with a peak modulus of 473 kPa under unconfined compression as well as exhibiting relaxation rates similar to those for native cartilage (Castilho et al., 2019). Controlling the mechanical properties of scaffolds for osteochondral TE is essential, not only to maintain structural integrity and withstand high mechanical loading *in vivo*, but also to provide environmental mechanical cues to selectively guide stem cell differentiation into the appropriate osteochondral phenotypes.

## Mechanical Cues Affecting Stem Cell Differentiation

MSC commitment to the chondrocytic lineage is governed by TGF-β and WNT/ß-catenin signalling (Usami et al., 2016). In particular, the activation of TGF-β/SMAD2/3 pathways is essential for the intracellular phosphorylation of Smad2 and Smad3, which then translocate to the nucleus to activate and stabilise the transcription factor Sex determining region Y (SRY) Box 9 (SOX9), that is the master regulator of chondrogenesis (Furumatsu et al., 2009; Coricor and Serra, 2016; Pfeifer et al., 2019). SOX9, along with SOX5 and SOX6 expression is required during embryonic development as well as in post-natal maintenance of articular cartilage regulating expression of ECM molecules, such as collagen (mainly types II, IX, XI) and proteoglycans (aggrecan, decorin).

**Figure 3:**
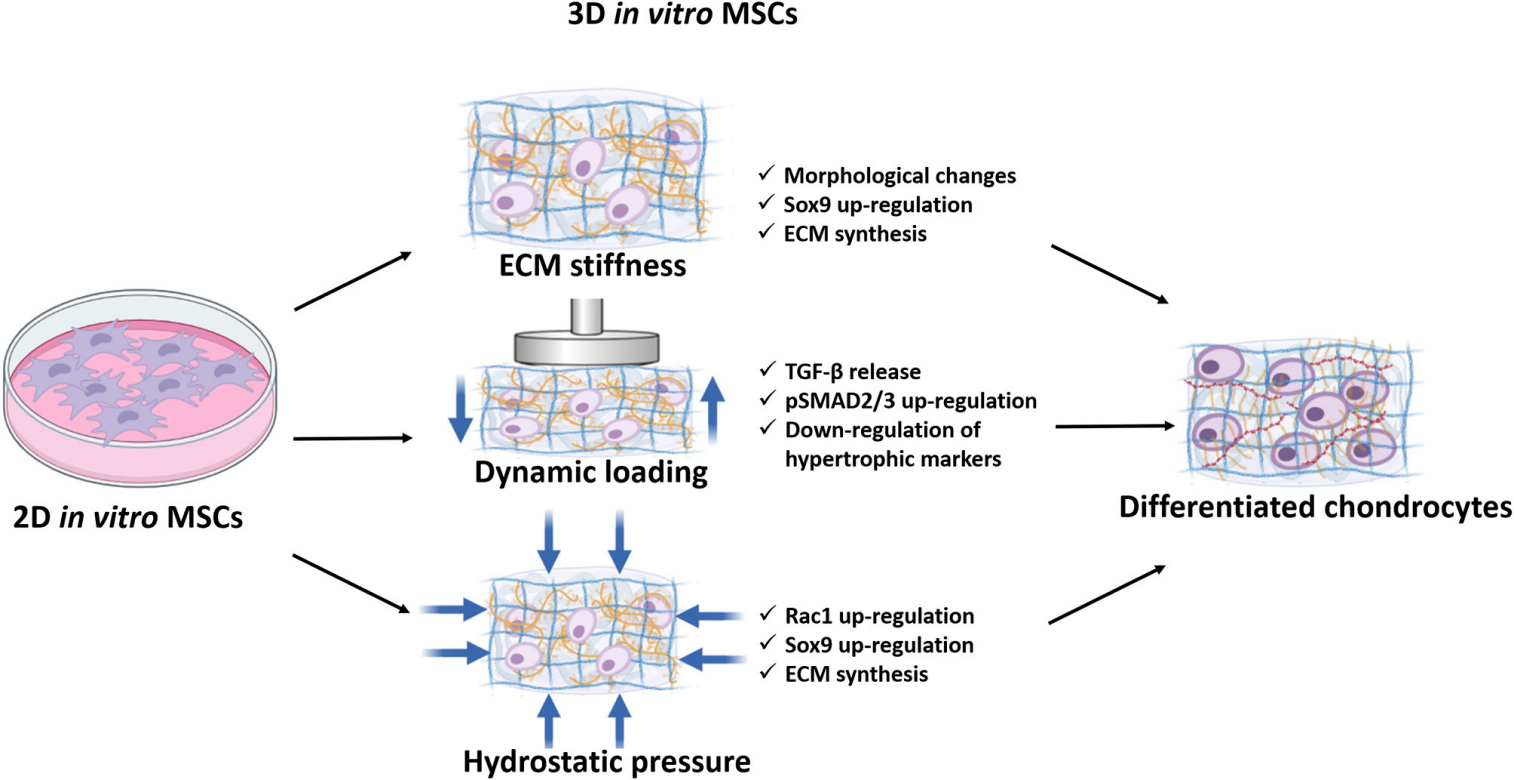
Schematic representation of the mechanical cues affecting stem cell differentiation down the chondrogenic lineage. MSCs are mechanosensitive in response to ECM stiffness, dynamic loading, and hydrostatic pressures which activates various signalling pathways necessary to drive differentiation down the chondrocytic lineage.

To differentiate MSCs into chondrocytes the use of growth factors, such as TGF-β, is usually required. However, its use in the clinic is limited as it leads to the expression of hypertrophic markers such as Col X, MMP13 and alkaline phosphatase (ALP), which will eventually lead to cartilage mineralization. There is increasing evidence that environmental (such as low oxygen tension) and mechanical cues control stem cell fate. In particular (as described in Figure 3), MSCs are highly mechanosensitive and respond to both passive stimuli such as stiffness, and dynamic stimulation such as mechanical loading and hydrostatic pressure that signals through integrins and focal adhesion (FA) protein complex, transducing physical signals into biochemical signals.

### Regulation of MSCs Differentiation by ECM Stiffness

*In vivo* each tissue is characterised by a specific stiffness, which regulates tissue development and homeostasis by affecting cell migration, proliferation, morphology, cell phenotype and ECM protein production (Ehrbar et al., 2011; Handorf et al., 2015; Hwang et al., 2015; Du et al., 2016; Sun et al., 2017; Xia et al., 2017; Abbas et al., 2019; Chu et al., 2019; d'Angelo et al., 2019; Saidova and Vorobjev, 2020). Engler et al. (2006) originally explored the effect of stiffness on MSCs using polyacrylamide hydrogels mimicking the native elasticity of brain, muscle and osteoid. This work demonstrated that stiffness not only affects MSC morphology showing that the expression of the neurogenic marker ß tubulin 3 was enhanced on soft substrates and Runx2 on stiff substrates. Interestingly, this work showed that growth factors tend to be less selective compared to matrix stiffness in driving lineage specification. MSCs pre-conditioned on a matrix with a specific stiffness for 3 weeks cannot be reprogrammed, suggesting that modulation of ECM modulus could be a powerful tool to drive stem cell differentiation. When cultured on stiff substrates, MSCs develop a highly organised cytoskeleton showing a spindle-shape morphology (Table 3). Conversely, MSCs on soft substrates exhibit a rounded chondrocyte-like morphology and express higher levels of chondrogenic markers. Park et al. (2011) compared collagen type II and GAG synthesis on a soft collagen hydrogel, on plastic coated with a thin layer of collagen and on polyacrylamide hydrogels with different stiffnesses (1 and 15 kPa). They showed an increase in expression of chondrogenic markers both on the soft collagen hydrogel and on the 1 kPa substrate. The effect of substrate stiffness together with biochemical cues was investigated by Wang et al. (2016). They showed that HA enhanced MSC chondrogenesis, evidenced by upregulated of aggrecan and Col II expression and this effect was more distinct when cells were grown in soft hydrogels (3 kPa), while this effect was reversed in the stiff hydrogel (90 kPa). It is important to note that cartilage stiffness varies between 0.1 and 6.2 MPa, and soft hydrogels will fail to maintain their structural integrity after implantation (Wang et al., 2016; Zheng et al., 2019; Guimarães et al., 2020). Olivares-Navarrete et al. (2017) compared both cytoskeletal organisation and gene expression of MSCs and auricular chondrocytes grown on methyl acrylate/methyl methacrylate (MA/MMA) polymer surfaces with elastic moduli ranging from 0.8 to 310 MPa mimicking the stiffness of articular cartilage and cortical bone. MSCs appeared to be elongated on the less stiff surfaces with a higher number of adhesion plaques on the 4.7 MPa substrate. After 7 days without exogenous stimuli by cytokines or other factors associated with cartilage differentiation, the expression of SOX9, Col II, aggrecan and cartilage oligomeric matrix protein (COMP) in MSCs showed an increasing trend with decreasing stiffness. This work showed that mimicking the native elasticity of cartilage enhances chondrogenic phenotype without exogenous stimuli. Nevertheless, it is also important to consider that osteochondral tissue exhibits different stiffness among the different layers, and an implant displaying a layering or gradient approach with varying stiffness, might be more effective in reproducing the native architecture of the tissue as well as selectively promoting ECM synthesis.

**Table 3:** Summary of the effect of mechanical cues on MSCs differentiation.

**EFFECT OF MECHANICAL CUES ON MSCs**				
**Substrate stiffness**	**Soft substrates (1–30 kPa)**	**Intermediate substrate (0.1–5MPa)**	**Stiff substrates (5–300 MPa)**	**References**
	MSCs on soft substrates exhibit a rounded chondrocyte-like morphology Higher expression levels of chondrogenic markers Unable to withstand mechanical loads	Mechanically competent Stiffness in the range of native cartilage MSCs found to express high level of SOX9 and Col II on substrates stiffness of 0.8MPa and 4.7MPa	Highly organised cytoskeleton Spindle-shape morphology	Engler et al., 2006; Park et al., 2011; Wang et al., 2016; Olivares-Navarrete et al., 2017
**Dynamic loading**	**0.15–1.5% compressive strain 1Hz**	**10% compressive strain 1 Hz from day 0**	**10% compressive strain 1 Hz after 1 week of pre-culture**	
	Dynamic loading, delayed osteogenesis. Mineral deposits was diffuse in the unloaded condition while under dynamic loading was concentrated and spatially restricted to the central region	Compression from day 0 has negative effects on MSC chondrogenesis	Dynamic culture increase synthesis of GAG aggrecan, Col II and increase expression SOX9 Upregulation of phosphorylated-SMAD2/3 MSCs under static culture MSCs exhibited higher of hypertrophic markers	Thorpe et al., 2010; Zhang et al., 2015; Sawatjui et al., 2018; Aziz et al., 2019; Cao et al., 2019
**Hydrostatic pressure**	**Low HP stimulation** **100–300 kPa**	**Physiological HP stimulation in the cartilage layer** **1–10 MPa**	**High HP** **25 MPa**	
	IHP upregulate osteogenic markers Increase expression of Runx2, ALP and osteopontin	HP applied continuously it negatively affects SOX9, Coll II and aggrecan gene expression IHP positively affects SOX9 and Col II expression even without external growth factors and enhances cartilaginous matrix deposition	Inhibited aggrecan and Col II Pro-osteoarthritic effects	Correia et al., 2012; Li et al., 2016; Montagne et al., 2017; Stavenschi et al., 2018; Stavenschi and Hoey, 2019

### Role of Dynamic Loading in MSCs

During ambulation mechanical load plays an important role in maintenance and degeneration of articular cartilage affecting gene expression of Col II, aggrecan, and degenerative enzymes (MMPs). Interestingly dynamic stimulation also affects MSC differentiation and the quality of ECM synthesised. A study from Thorpe et al. (2008) revealed a negative effect of long term dynamic compression on MSCs cultured in agarose hydrogels. This study reported that unconfined compression at 10% strain and 0.5 Hz for 1 h/day significantly reduced GAG production and Col II synthesis compared to static culture. Interestingly, the application of dynamic compression from day 0 inhibits chondrogenesis even in the presence of TGF-β3 (Thorpe et al., 2010). In contrast, the inhibition of chondrogenesis in response to dynamic compression was not observed if the MSCs were first allowed to undergo chondrogenesis. Consistent with these results, Sawatjui et al. (2018) studied the effect of dynamic compression of both MSCs and chondrocytes derived from osteoarthritic joints seeded on silk fibroin scaffold, pre-cultured for 1 week, and subsequently subjected to compression with 10% dynamic strain at 1 Hz, 1 h/day for 2 weeks. This study showed that dynamic compression significantly enhanced the synthesis of Col II and aggrecan along with an increase of compressive modulus. Cao et al. (2019) seeded rabbit derived MSCs into collagen scaffolds under 10% compressive sinusoidal strain at 1 Hz frequency, for 2 h/day for 21 days. Starting from the second week of culture, the morphology of MSCs in the dynamic culture group exhibited a rounded chondrocyte-like morphology, whereas cells remained spindle shaped in static culture. Dynamic culture also promoted GAG synthesis as well as aggrecan, Col II and SOX9 expression compared to the static culture. Zhang et al. (2015) demonstrated that delayed dynamic compression positively affected MSC chondrogenesis through phosphorylated-SMAD2/3 enhancing matrix deposition and suppressing hypertrophy. Further MSCs under free swelling condition exhibited higher expression of ERK (involved in chondrocyte hypertrophy) along with upregulation of MMP13, Runx2, and Col X. In addition, Gardner et al. (2017) demonstrated that multiaxial loads on MSC led to endogenous production and secretion of TGF-β1 as well as the activation of the secreted latent TGF-β1. Taken together these data suggest that dynamic load positively affects MSC chondrogenesis, however, MSCs should first be differentiated before applying loads. Consequently *in vitro* differentiation of stem cells prior to implantation could be critical for osteochondral tissue engineering.

### Hydrostatic Pressure and MSCs Differentiation

Cartilage ECM is characterised by a high water content and low permeability, and as a consequence when a load is applied the resistance of fluid flow generates hydrostatic pressure (HP). *In vivo* HP varies between 2 and 10 MPa with peaks of 18 MPa during intense activities such as jumping or running (Elder and Athanasiou, 2009; Correia et al., 2012). Several studies have demonstrated that the application of HP on MSCs might have a pro-chondrogenic effect. Angele et al. (2003) examined the effects of cyclic hydrostatic pressure on MSCs aggregates showing a significant increase in GAG and collagen content at days 14 and 28 compared to the unloaded control. Furthermore, Miyanishi et al. (2006a) studied MSCs in pellet culture exposed to intermittent hydrostatic pressure (IHP) and demonstrated an increase in expression of SOX9, Col II, and aggrecan with or without the addition of TGF-β3. In a second study the authors also demonstrated that the magnitude of loading modulated chondrogenic gene expression and cartilage matrix protein deposition in MSCs pellets in the presence of TGF-β3 suggesting that the magnitude of the load could enhance MSCs chondrogenesis (Miyanishi et al., 2006b). In fact, physiological levels of HP (5MPa) significantly enhance cartilaginous matrix deposition (Correia et al., 2012; Li et al., 2016). Conversely, high HP (25 MPa up to 24 h) on the ATDC5 cell line markedly affecting the expression of matrix remodelling related genes, apoptosis-related genes and strongly inhibited aggrecan and Col II, suggesting that excessive loads induce pro-osteoarthritic effects (Montagne et al., 2017). Interestingly the use of low HP, in the range of 100–300 kPa, has been demonstrated to direct MSCs differentiation into the osteogenic lineage upregulating the expression of Runx2, ALP and osteopontin (Stavenschi et al., 2018; Stavenschi and Hoey, 2019). Not only the magnitude of load, but also the length of the stimulation affects matrix deposition. In fact, it has been shown that when the load is applied continuously, it negatively affects SOX9, Col II and aggrecan gene expression (Correia et al., 2012; Li et al., 2016).

One of the major limitations of cartilage tissue engineering is the formation of fibrocartilage, which has inferior mechanical properties compared to articular cartilage. HP appears to affect hypertrophic genes, increasing Col I, Col X and MMP13 (Ogawa et al., 2009, 2015; Li et al., 2016). Conversely, other studies revealed decreasing levels of Col I and Col X under IHP (Vinardell et al., 2012; Saha et al., 2017; Rieder et al., 2018). Freeman et al. (2017) demonstrated that HP without any external growth factors resulted in enhanced chondrogenesis along with reduction in hypertrophic markers. Additionally, when MSCs were stimulated with HP alone and subsequently induced to undergo osteogenic differentiation without any external mechanical stimulation, the production of hypertrophic markers was reduced compared to those exposed to chondrogenic growth factors alone. These studies suggested that the application of intermittent hydrostatic pressure could potentially lead to a stable differentiation of MSCs into the chondrogenic lineage without the use of growth factors. However, it is important to note that the intensity and the frequency of HP applied differ among studies, suggesting that standardisation is required to obtain consistent results.

## Testing Osteochondral Graft Materials

The success of osteochondral grafts depends on the restoration of surfaces representative of native articular cartilage to provide smooth joint movement during joint articulation. Implanted grafts also need to be structurally stable to withstand physiological loading conditions of up to 4–5 times body weight during walking (Morrison, 1970; Bellucci and Seedhom, 2001) with peak stresses in the knee ranging from 2 to 10 MPa and at a loading frequency of approximately 1 Hz (Brand, 2005; Sadeghi et al., 2015). Osteochondral defects cause high contact stresses at the rim, that vary depending on the size of the defect causing uneven strain distribution (Brown et al., 1991; Kock et al., 2008). These abnormal contact stresses and strains at the defect perimeter cause damage and chondrocyte death that could impair integration and healing of the graft, leading to reduced functionality of the joint, or cartilage damage (D'Lima et al., 2001; Wu et al., 2002). However, contact stresses can be restored to pre-operative levels, resembling intact cartilage depending on appropriate fitting, alignment, length and surface of the graft (D'Lima et al., 2001; Koh et al., 2004; Kock et al., 2006, 2008). One of the major issues is that post-implantation osteochondral implants will be subject to continual cyclic loads, encompassing a range of shear and tensile forces which will affect the biological response of the graft and test the integration with the surrounding native cartilage. However, specific test methods to demonstrate the performance of these grafts have not yet been defined.

### Standardisation

Osteochondral grafts are classed by the International Standard Organisation (ISO) as implantable medical devices that as defined in ISO 13485, 2016, are implanted into the human body via surgical intervention and are intended to remain in place after the procedure. ISO 14630:2012 specifies the general requirements for non-active surgical implants, whereas ISO 21536:2007 is the level 3 standard referring more specifically to knee joint replacement implants. These standards include performance, design, materials, evaluation and sterilisation and the tests needed to demonstrate compliance with these regulations. More specific standards relating to tissue engineered cartilage constructs include the quantification of sulfated glycosaminoglycans (sGAG) (ISO 13019: 2018), and the evaluation of tissue morphology including collagen fibre orientation and anisotropy *in vivo* (ISO/TR 16379:2014) have also been defined. Despite these biological and clinical evaluation there are no specific requirements for mechanical testing, and there is uncertainty as to whether articular cartilage implants are classified as partial joint replacement implants and should therefore be subject to mechanical characterisation (Marchiori et al., 2019).

In contrast, the FDA provides more specific mechanical testing criteria for the use of tissue engineered cartilage constructs, which highlights inconsistencies with regard to global standardisation. The FDA guidance document for products intended to repair or replace knee cartilage includes specifications for *in vivo* animal studies (that will be discussed later in this review) and various *in vitro* mechanical tests. It states that “mechanical testing should address the following: the ability of the implant to withstand expected *in vivo* static and dynamic loading (i.e., compression, shear, and tension); analysis of fixation method (i.e., strength of integration between the product and surrounding native tissue); and propensity to generate wear debris.” It is also recommended that static mechanical behaviour such as the maximum recoverable compressive strain, the aggregate modulus (HA), the shear modulus (G), and permeability (κ) as well as the dynamic complex shear modulus are included. Degradable scaffolds should also include assessment of failure properties over time and some examples of confined or unconfined compression and indentation are suggested for analysing the mechanical properties of implants.

### *In vitro* Compressive Testing (Confined, Unconfined and Indentation)

**Figure 4:**
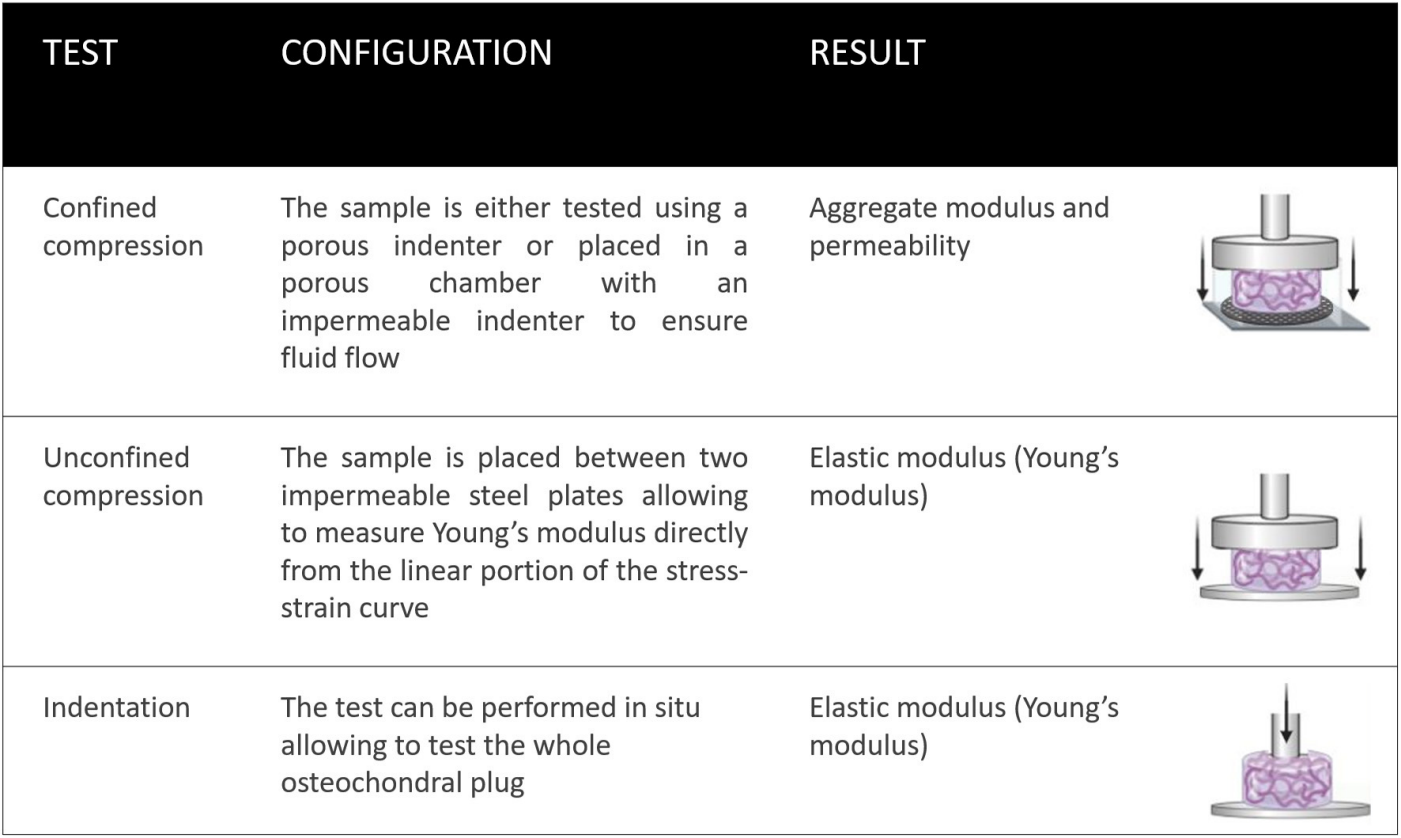
Standard mechanical tests for assessing osteochondral grafts. Confined compression tests using either an impermeable chamber and porous indenter; or porous chamber with an impermeable indenter, are useful for defining the aggregate modulus. Unconfined compression tests and indentation tests can determine the elastic modulus (Young's modulus).

The most frequent *in vitro* test are usually biological assays to evaluate the biocompatibility (ISO 1099), cytotoxicity (ISO 10993-5), gene expression and matrix deposition (ISO 13019 quantification of sulfated glycosaminoglycans for evaluation of chondrogenesis) (Keong and Halim, 2009; Li W. et al., 2015). However, mechanical evaluation of osteochondral scaffolds are essential to ensure graft stability in the initial period following implantation (Bowland et al., 2015). As reviewed by Patel et al. (2019), compression testing is the most common test performed both on cartilage and tissue engineered construct. Compression test can be performed using unconfined and confined compression and indentation (Figure 4). For unconfined compression testing, the sample is placed between two impermeable steel plates allowing the Young's modulus to be measured directly from the linear portion of the stress-strain curve produced (Korhonen et al., 2002; Griffin et al., 2016). For confined compression the sample is either tested using a porous indenter or placed in a porous chamber with an impermeable indenter to ensure fluid flow. Confined compression allows the measurement of both the aggregate modulus (determined when fluid flow stops) of the specimen as well as the permeability (Boschetti et al., 2004). While unconfined and confined compression require the cartilage sample or the scaffold to be tested within a chamber, indentation allows the test to be performed on a whole osteochondral specimen (Griffin et al., 2016; Tozzi et al., 2020). Compression tests can be performed by applying a strain at a constant rate (ramp), by applying a strain to a target level and holding the strain constant (stress-relaxation) or applying a cyclic strain (dynamic) (Scholten et al., 2011; Vikingsson et al., 2015; Coluccino et al., 2016; Kundanati et al., 2016). Compression tests can be also load-controlled, applying a rapid load that is then kept constant, measuring sample strain over time (Oyen, 2014; Patel et al., 2019). Both the FDA and International Cartilage Repair Society (ICRS) recommend both static and dynamic compression tests to assess the mechanical behaviour of the osteochondral graft. However, specific guidelines on how to perform each test have not been agreed, which leads to inconsistent or non-physiologically relevant data. Cartilage and osteochondral grafts should be tested under the same conditions, as the strain rate influences the stress-strain curves, implying that the higher the strain rate the higher the modulus will be. As reviewed by Patel et al. (2019), 48% of the studies that analysed cartilage repair constructs under ramp mechanical testing, were tested to more than 20% strain, more than double the compressive strain that articular cartilage was tested to. Considering that the physiological average strain is 10% the data produced using higher strain might not be reliable (Chan et al., 2016).

In addition to the standard mechanical tests previously mentioned, implants need to be tested after periods *in vivo* (for dynamic and static loading) and under loading conditions of compression, tension, and shear. Analysis of fixation within the defect is also required (e.g., mechanical push-out tests to assess integration) and assessment of the bioreactivity of any device-generated wear debris.

### *In vivo* Animal Models

*In vivo* animal models are crucial preliminary studies to assess the safety and efficacy of newly developed cartilage TE implants. However, currently there are no exact guidelines for the comparison of animal models, assessment of defect size/location and description of appropriate mechanical tests for the assessment of implantable devices such as osteochondral grafts to repair and regenerate articular cartilage. The FDA recommends using combined animal studies with appropriate mechanical testing to assess biological response, durability (using large animal models) and toxicology (U. S. Food Drug Administration, 2011). In addition, dose response (of bioactive scaffolds), lesion size and location, appropriate endpoints, and continual arthroscopic/MRI evaluation should also be taken into consideration (U. S. Food Drug Administration, 2011). Nevertheless, despite these recommendations there are no clearly defined protocols, test criteria, or test parameters for mechanical testing. It is also acknowledged that there is no optimal animal model for cartilage repair, which may also lead to variability between studies.

Small animal models (mouse, rat and rabbit) are mainly used for “proof of concept” studies as a translational step between *in vitro* tests and larger animal/human studies. Rabbit models have spontaneous intrinsic healing capabilities of cartilage defects that must be taken into consideration, therefore, they usually require additional validation in other animal models (Shapiro et al., 1993). Other variables to consider when choosing the most appropriate animal model are thickness of cartilage and joint suitability, skeletal/ cartilage maturity, defect type, size and location of the defect, availability and post-operative care (Hurtig et al., 2011). Canine models, like humans, often suffer from diseases such as OA and OCD which makes them useful for assessing cartilage regeneration in pathologic conditions (Chu et al., 2010). Large animal models (goat, sheep, pig and horse) more closely reflect intended clinical use for assessing toxicity, integrity and inflammatory responses for both small and larger defects in load bearing environments. Since cartilage thickness in equine stifle joints (1.5–2.0 mm) is the most similar to human cartilage thickness (2.2–2.5 mm) the horse is the closest approximation for creating both partial and full thickness defects for preclinical cartilage repair studies (Frisbie et al., 2006; McIlwraith et al., 2011). Nevertheless, in most animal models the loading, thickness and geometry of the joint surface is very different to that of humans.

### Mechanical Push-Out Tests for Assessing Integration

Mechanical push-out tests are useful pre-clinical studies to evaluate the maximum forces needed for graft failure and for assessing integrative repair with host cartilage over time (Theodoropoulos et al., 2011). A biopsy punch is normally used to create a cylindrical defect filled with the TE scaffold or osteochondral graft to be tested. After a culture and/or treatment period to allow a certain amount of integration with the host tissue, the inner core is pushed out of the outer ring using a mechanical push-out rod. The calculated amount of force needed for displacement (or failure of the graft) allows an assessment of integrative strength at the interface to be assessed. A recent study by Bowland et al. (2020) performed a series of push-in and push-out tests to assess the mechanical stability of bovine and porcine osteochondral grafts. Interestingly, the results showed that the harvesting method (using a trephine drill or chisel) showed no significant differences in graft stability (Bowland et al., 2020). However, preparation of the recipient site, the depth of insertion and dilation had more of an effect, showing that grafts with equal lengths to the site of insertion were more stable, and that dilation of grafts reduces the stability particularly in more skeletally immature tissue (Bowland et al., 2020). This research also highlights the importance of the underlying subchondral bone and the interrelationship between these tissues on regeneration and durability of focal defects consistent with other studies (Chan et al., 2012).

### Whole Joint Simulation Models to Mimic Joint Articulation

In contrast, *in vitro* whole joint simulations can be used to assess the tribological performance of osteochondral grafts, taking into consideration the interactions and biomechanical properties of the joint as a whole under physiological loading conditions. These types of test are relevant for comparing the efficacy of osteochondral grafts to other surgical interventions such as scaffolds and cell-based approaches (Bowland et al., 2018b). Bowland et al. (2018a) used an adapted method from Liu et al. (2015) using a whole joint simulator with six degrees of freedom and five controlled axes of motion to mechanically test grafts. The axial load was force controlled, tibial rotation (1.6–1.6°) and flexion/extension (0–21°) were displacement controlled at a frequency of 1 Hz. Anterior-posterior displacement was constrained using springs that generated rolling and sliding motions of the femur against the tibia, and mimicked ligament function. The medial-lateral axis was fully constrained and abduction/adduction was under passive motion. The main finding of this study was that allograft plugs fitted flush with the defect site to restore the articular surface caused the least wear and damage on the opposing joint surface after applying a complex range of motions. Similarly, Nebelung et al. (2017) combined a whole-knee joint loading device with MRI imaging to non-invasively assess the structural and functional responses of human articular osteochondral grafts in defect sites during *in situ* compressive loading. Whole joint simulation models highlight the importance of restoring the congruence of articular surfaces during an experimental setting that mimics more closely the physiological environment of joint articulation. However, the use of cadaveric tissue with diluted serum replicating the joint's synovial fluid is a useful approach but it fails to replicate large numbers of walking cycles due to limitations regarding the continual sterility and viability of the tissue.

### Shear Stress to Assess Tribology

Chondrocytes in the superficial layer produce lubricin that maintains low coefficients of friction of joints. Maintaining a low frictional interface is essential to prevent mechanical wear and erosion of the articular surface. The application of frictional shear stress has been shown to cause damage such as cracking and peeling in cartilage TE constructs, which are not seen in native cartilage controls (Whitney et al., 2017). Therefore, assessing the tribology of osteochondral grafts is essential to ensure adequate integration and longevity. To measure the frictional coefficient, three different configurations of tribometer can be used: pin-on-disc, pin-on-plate, or rolling-ball-on-disc. In the first two settings a pin is glued to the sample and a disc or a plate are in motion, while for the rolling-ball-on-disc the disc and the ball can be moved independently. In each configuration a normal force is applied and a sensor measures the frictional force, the frictional coefficient can be derived by dividing the frictional force for the normal force applied. Different types of lubricant can be used (i.e., PBS or foetal bovine serum) which combined with the different testing configurations often lead to variable results, highlighting the need of standardisation procedure to test both cartilage samples and osteochondral TE constructs. Other mechanical tests such as frictional shear stress testing can assess the tribology, pressure distribution and the response to damage of osteochondral grafts and TE constructs in whole joint models under a complex range of sliding and torsional motions (Walter et al., 2013; Bobrowitsch et al., 2014).

## Conclusions

Despite tremendous advances in the field of tissue engineering, an optimal biomaterial system for osteochondral defects that is able to direct stem cell differentiation into chondrocytes for the cartilage and osteoblast for bone without the use of exogenous stimuli is elusive. Material selection is essential for creating a graft able to withstand the multiple forces that cartilage is subject to. Synthetic materials not only provide high tensile stress and compressive modulus, but they are easily modified, facilitating the creation of layered scaffolds which is a requirement for osteochondral grafts. However, the lack of cellular binding sites require them to be combined with natural materials, which are highly biocompatible and can provide biochemical cues for stem cell differentiation. The natural architecture of cartilage and the impermeable subchondral plate enhances the development of hydrostatic stress in the cartilage which promotes and maintains the chondrocytic phenotype, however few osteochondral implant designs replicate this sub-chondral barrier.

Although suitable mechanical properties are essential for ensuring graft stability *in vivo*, the optimal range of stiffnesses is yet to be determined. Conflicting results have been reported, as to whether high stiffness could enhance chondrogenic differentiation of MSCs or upregulate hypertrophic markers. The use of dynamic stimulation, such as hydrostatic pressure or dynamic loading, could promote a stable differentiation of MSCs into chondrocytes and enhance matrix deposition, thus preventing the use of TGF-β which lead to the formation of hypertrophic cartilage.

Mechanical testing of TE constructs *in vitro* are essential to ensure graft stability *in vivo*, however, the lack of standardised procedures questions the reliability of the published data in providing an understanding of the long term endurance and suitability of osteochondral grafts. In addition, only a small fraction of studies on cartilage constructs tests all of the mechanical properties requested from the FDA or the ICRS and this might, in part, explain why many scaffolds fail when tested *in vivo*.

## Author Contributions

SD and TR completed a literature search, wrote the manuscript and critically revised the manuscript. MR, GB, and GT contributed ideas, content to the manuscript, and critically revised the manuscript. All authors contributed to the article and approved the submitted version.

## Conflict of Interest

The authors declare that the research was conducted in the absence of any commercial or financial relationships that could be construed as a potential conflict of interest.
